# Tyndallized Bacteria Preferentially Induce Human Macrophage M1 Polarization: An Effect Useful to Balance Allergic Immune Responses and to Control Infections

**DOI:** 10.3390/antibiotics12030571

**Published:** 2023-03-14

**Authors:** Serena Di Vincenzo, Maria Ferraro, Simona Taverna, Velia Malizia, Marco Buscetta, Chiara Cipollina, Valentina Lazzara, Paola Pinto, Marco Bassano, Stefania La Grutta, Elisabetta Pace

**Affiliations:** 1 Institute of Translational Pharmacology (IFT)—National Research Council (CNR), 90100 Palermo, Italyelisabetta.pace@ift.cnr.it (E.P.); 2 Rimed Foundation, 90100 Palermo, Italy; 3 NBFC—National Biodiversity Future Center, 90100 Palermo, Italy; 4 Dipartimento Promozione della Salute, Materno-Infantile, di Medicina Interna e Specialistica di Eccellenza “G. D’Alessandro” (PROMISE), Università degli Studi di Palermo, 90100 Palermo, Italy; 5 Dipartimento di Farmacia, Università degli Studi-Federico II, 80100 Napoli, Italy

**Keywords:** macrophages, polarization, postbiotics, immune responses

## Abstract

Macrophage polarization is a dynamic process through which macrophages acquire specific features whose extremes are represented by M1 and M2 polarization. Interleukin (IL)-6, IL-1β, IL-12 and IL-8 belong to M1 macrophages while transforming growth factor-beta (TGF-β belongs to M2 cytokines. M2 polarization prevalence is observed in allergic diseases. Tyndallization is a thermal process able to inactivate microorganisms and to allow their use for chronic respiratory disease treatment via immune response modulation. The present study explores the effects of a blend of tyndallized bacteria (TB) on macrophage polarization. THP-1-derived macrophages were exposed to different concentrations of TB (10^6^, 5 × 10^6^, 10^7^, 5 × 10^7^, 10^8^ CFU/mL) and then cell viability and TB phagocytosis, and IL-8, IL-1β, IL-6, IL-12 and TGF-β1 gene expression and release were assessed. TB were tolerated, phagocyted and able to increase IL-8, IL-1β and IL-6 gene expression and release IL-12 gene expression, as well as decrease TGF-β1 gene expression and release. The effects on IL-8, IL-6 and TGF-β1 release were confirmed in human monocyte-derived macrophages (hMDMs) exposed to TB. In conclusion, TB promote M1 polarization, and this mechanism might have valuable potential in controlling allergic diseases and infections, possibly preventing disease exacerbations.

## 1. Introduction

Macrophages represent 70% of the immune cells in the lung [1]. These cells exert a crucial role in the regulation of airway inflammation in allergic diseases of the upper (rhinosinusitis) and lower (asthma) airways since they can promote both pro- and anti-inflammatory responses through the release of signaling mediators [1]. It has been well established that macrophages are a heterogeneous innate immune cell population undergoing a process known as cell polarization [2]. Macrophage polarization is a dynamic process by which a macrophage expresses different functional phenotypes in response to different micro-environmental signals. The extremes of the macrophage polarization are represented by the classically activated (M1) and alternatively activated (M2) macrophages. Different subtypes of M2 are described: M2a, M2b, M2c and M2d. M2a is induced by IL-4 and IL-13, M2b is induced by immune complexes, M2c is induced by anti-inflammatory cytokines (i.e., IL-10) or glucocorticoids, and M2d is induced by IL-6–like cytokines [3]. Differently, microbial components and Interferon (IFN)-gamma induce M1 polarization. M1 macrophages preferentially drive inflammation to eliminate intracellular pathogens and are associated with T helper (TH) 1 responses [4]. M2 macrophages regulate wound healing, the clearance of dead cells and anti-inflammatory responses, and are associated with TH2 responses [5]. M1 cells produce inflammatory cytokines including IL-1β, IL-12, IL-6 and chemokines including IL-8, while M2 cells are characterized by the secretion of IL-10 or TGF-β [5] other than the production of extracellular matrix components, angiogenic and chemotactic factors [6].

A balanced proportion of M1 and M2 preserves the immune system by dysregulated immune reactions, i.e., autoimmune (prevalence of M1 polarization) [4] or allergic (prevalence of M2 polarization) [7] responses. M2 and M1 macrophages can interconvert with the M1 or M2 phenotype in response to micro-environmental signals that also involve the activation of Toll-like receptors (TLRs). TLRs are representative pattern-recognition receptors (PRRs) with important roles in the recognition of microbe-associated molecular patterns. Lipoteichoic acid (LTA) and lipoproteins of Gram-positive bacteria are ligands of TLR2 and are a potent inducer of TNF-α, IL-8 and IL-1β release in PMA-differentiated THP-1 cells [8]. TLR2 activation by probiotic bacteria can lead to anti-inflammatory states that improve the mucosal barrier integrity [9]. THP-1 cells (human leukemia monocytic cell line) stimulated with PMA constitutes a suitable model for studying macrophage polarization in vitro [10].

Probiotics are considered non-pathogenic live microorganisms that, when administered in adequate amounts, exert beneficial effects [11]. Increased interest towards the use of non-viable heat-killed probiotics is growing due to safety issues, especially regarding the use of live probiotics in vulnerable populations, such as immunocompromised patients or neonates [12].

Postbiotics obtained by heat treatment can release bacterial components with key immunomodulating, anti-inflammatory and mucosal protective effects [13]. Tyndallized bacteria (TB) are obtained with a combination of heat (70–100 °C) treatments with incubation periods at lower temperatures [13]. The use of heat-inactivated probiotics (postbiotics) improved allergen-induced airway inflammation symptoms by modulating the host immune response toward TH1 dominance [14,15]. Limited information is available on the effects of postbiotics, including TB, on macrophage polarization.

The present study was aimed to assess the immunomodulatory effect of a TB blend composed of *Lactobacillus Casei*, *Lactobacillus Acidophilus*, *Lactobacillus Plantarum*, *Streptococcus Thermophilus* on M1/M2 polarization of THP-1 derived macrophages cell line as well as of primary human monocyte-derived macrophages (hMDMs).

## 2. Results

### 2.1. Effect of TB on the Viability of THP-1 Derived Macrophages

**Figure 1:**
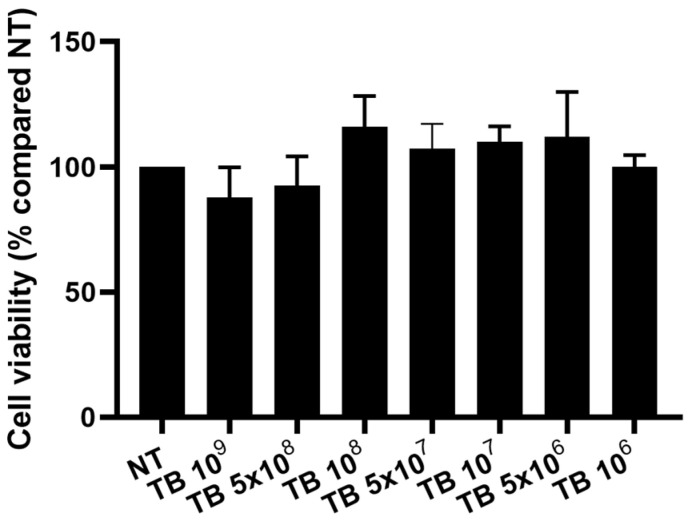
Effect of TB on the viability of THP-1-derived macrophages. THP-1-derived macrophages were incubated with different concentrations of TB and the effect on the viability was assessed by MTS assay. Data are expressed as mean ± SD (N = 3).

Cell viability of THP-1 derived macrophages treated with different concentrations of TB (10^6^, 5 × 10^6^, 10^7^, 5 × 10^7^, 10^8^, 5 × 10^8^, 10^9^ CFU/mL) was evaluated by MTS assay. Although TB concentrations at all tested concentrations did not significantly decrease cell viability (Figure 1), for the subsequent experiments, we preferred to use 10^6^, 5 × 10^6^, 10^7^, 5 × 10^7^, 10^8^ because the highest concentrations (5 × 10^8^ and 10^9^ CFU/mL) tend to decrease cell viability.

### 2.2. Effect of TB on the Phagocytosis of THP-1-Derived Macrophages

**Figure 2:**
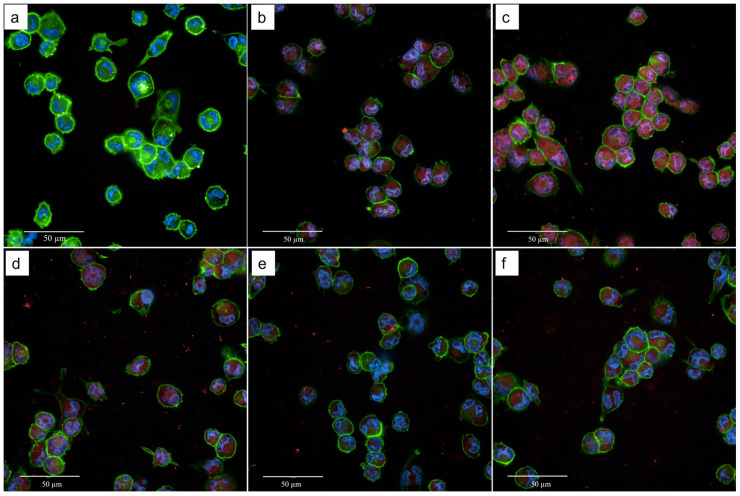
Effect of TB on the phagocytosis in THP-1-derived macrophages. THP-1-derived macrophages were incubated for 30 min without (**a**) or with different concentrations of TB ((**b**) = 10^8^; (**c**) = 5 × 10^7^; (**d**)= 10^7^, (**e**) = 5 × 10^6^, (**f**) = 10^6^) and the effect on phagocytosis was evaluated. Representative images (one of three replicates) are shown (Blue: Hoechst, Green: actin, Red: TB) (**a**–**f**). Scale bar 50 µm.

Since it is well-known that phagocytosis activates macrophages [16] and enforces innate immune responses [17], we tested whether TB were phagocyted by THP-1-derived macrophages. As shown in Figure 2, THP-1-derived macrophages quickly phagocyted TB as confirmed by red fluorescence present in the cytoplasm.

### 2.3. Effect of TB on the Gene Expression and Release of Cytokines Associated with M1 Polarization (IL-8, IL-1β, IL-6 and IL-12) by THP-1-Derived Macrophages

**Figure 3:**
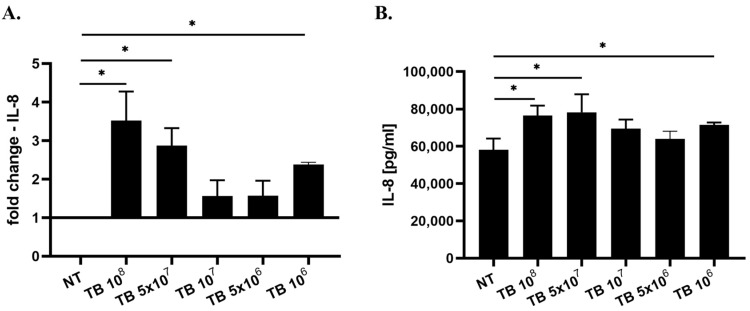
Effect of TB on the gene expression and release of IL-8 by THP-1-derived macrophages. THP-1-derived macrophages were incubated for 4 h with different concentrations of TB and the effect on the gene expression and protein release of IL-8 was assessed by Real Time PCR (**A**) and ELISA (**B**), respectively. Data are expressed as mean ± SD (N = 4). For statistical analysis, repeated measures ANOVA with Bonferroni post hoc test was applied. * *p* < 0.05 vs. NT.

IL-8 is the most abundantly secreted M1 cytokine [18]. Dose–response effects of TB gene expression and protein release of IL-8 were evaluated. The incubation of THP-1-derived macrophages with TB significantly increased IL-8 gene expression and release at 10^8^, 5 × 10^7^ and 10^6^ CFU/mL (Figure 3A,B). These TB concentrations were selected for subsequent experiments.

**Figure 4:**
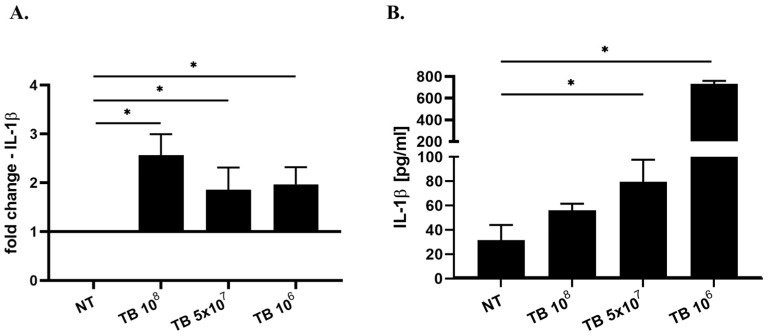
Effect of TB on the gene expression and release of IL-1β by THP-1-derived macrophages. THP-1-derived macrophages were incubated for 4 h with different concentrations of TB and the effect on the gene expression and protein release of IL-1β was assessed by Real Time PCR (**A**) and ELISA (**B**), respectively. Data are expressed as mean ± SD (N = 4). For statistical analysis, repeated measures ANOVA with Bonferroni post hoc test was applied. * *p* < 0.05 vs. NT.

Phagocytosis represents a crucial and fundamental event for the assembly of canonical and non-canonical inflammasome activation that leads to IL-1β release [19]. The effect of TB on the gene expression and release of IL-1β by THP-1-derived macrophages was tested. As shown in Figure 4, TB increased both gene expression at all the tested concentrations of TB (Figure 4A) and the release of mature IL-1β (Figure 4B) at 5 × 10^7^ and 10^6^ CFU/mL of TB in THP-1-derived macrophages.

**Figure 5:**
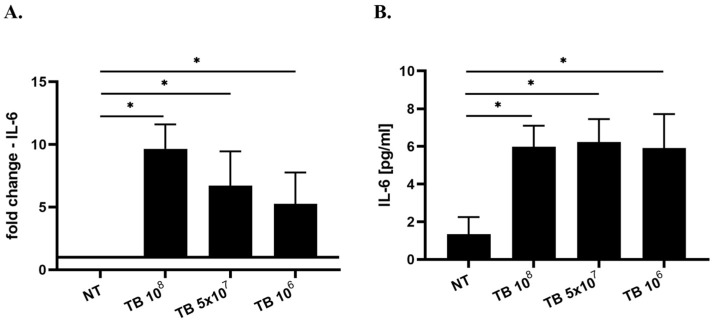
Effect of TB on the gene expression and release of IL-6 by THP-1-derived macrophages. THP-1-derived macrophages were incubated for 4 h with different concentrations of TB and the effect on the gene expression and protein release of IL-6 was assessed by Real Time PCR (**A**) and ELISA (**B**), respectively. Data are expressed as mean ± SD (N = 4). For statistical analysis, repeated measures ANOVA with Bonferroni post hoc test was applied. * *p* < 0.05 vs. NT.

IL-6 is also typically associated with M1 macrophages [20]; however, despite this it is considered a pleiotropic cytokine that can contribute both to the M1 or M2 polarization since it enhances the phenotype to which a macrophage has committed [21]. All the tested concentrations of TB significantly increased IL-6 gene expression (Figure 5A) and IL-6 protein release (Figure 5B) in THP-1-derived macrophages.

**Figure 6:**
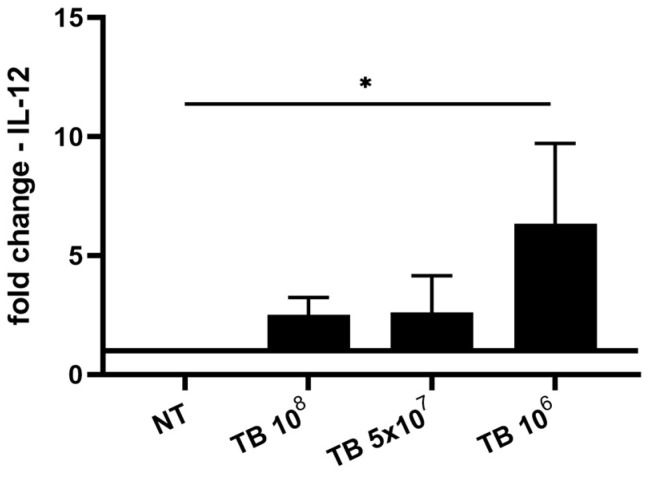
Effect of TB on the gene expression of IL-12 by THP-1-derived macrophages. THP-1-derived macrophages were incubated for 4 h with different concentrations of TB and the effect on the gene expression of IL-12 was assessed by Real Time PCR. Data are expressed as mean ± SD (N = 4). For statistical analysis, repeated measures ANOVA with Bonferroni post hoc test was applied. * *p* < 0.05 vs. NT.

Pattern-recognition receptors (PRR) stimulated by pathogen-associated molecular patterns (PAMPs) are able to induce IL-12 expression [22]. TB at 10^6^ CFU/mL significantly increased IL-12 gene expression (Figure 6).

### 2.4. Effect of TB on the Gene Expression and Release of Cytokines Associated with M2 Macrophages (TGF-β1) by THP-1-Derived Macrophages

**Figure 7:**
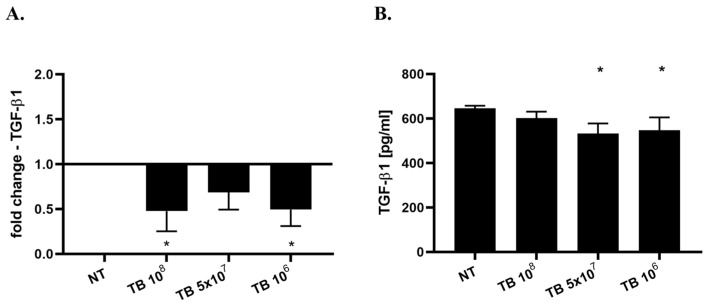
Effect of TB on the gene expression and release of TGF-β1 by THP-1-derived macrophages. THP-1-derived macrophages were incubated for 4 h with different concentrations of TB and the effect on the gene expression and protein release of TGF-β1 was assessed by Real Time PCR (**A**) and ELISA/(**B**), respectively. Data are expressed as mean ± SD (N = 4). For statistical analysis, repeated measures ANOVA with Bonferroni post hoc test was applied. * *p* < 0.05 vs. NT.

M2 macrophages are characterized by the production of anti-inflammatory cytokines, including TGF-β1, which contribute to tissue repair and remodeling [20]. The effects of TB on TGF-β1 gene expression and protein release were then tested in THP-1-derived macrophages. TB significantly down-regulated TGF-β1 gene expression at the concentrations of 10^8^ and 10^6^ (Figure 7A), while they significantly down-regulated TGF-β1 protein release at the concentrations of 5 × 10^7^ and 10^6^ CFU/mL (Figure 7B).

### 2.5. Effect of TB on the Release of IL-8, IL-6, IL-1β and TGF-β1 by hMDMs

**Figure 8:**
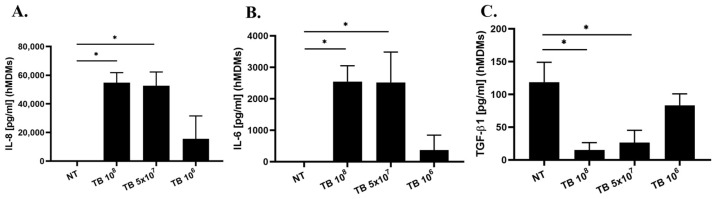
Effect of TB on the release of (**A**) IL-8, (**B**) IL-6 and (**C**) TGF-β1 by hMDMs. hMDMs were incubated with the indicated concentrations of TB for 4 h, and the effect on the protein release of IL-8, IL-6 and TGF-β1 was assessed by ELISA. Data are expressed as mean ± SD (N = 5). For statistical analysis, repeated measures ANOVA with Bonferroni post hoc test was applied. * *p* < 0.05 vs. NT.

In order to increase the physiological relevance of our data, the impact of TB on hMDM polarization was also evaluated. We assessed whether TB modified the release of IL-8, IL-6, IL-1β and TGF-β1 in hMDMs. As shown in Figure 8, TB were able to significantly induce IL-8 (Figure 8A) and IL-6 (Figure 8B) release and decrease TGF-β1 (Figure 8C) release in hMDMs.

## 3. Discussion

The increased incidence of allergic diseases can be due to alterations in the composition of the airway *microbioma* due to a reduced exposure to beneficial symbiotic bacteria or parasites [23]. In animal models of soybean allergy, *Lactobacillus Acidophilus*, *Lactobacillus Delbrueckii subsp*, *Bulgaricus* and *Lactobacillus Plantarum* are able to alleviate the allergic symptoms by reducing the production of TH2 cytokines and IgE while increasing TH1 cytokines and T regulatory (Treg) lymphocytes [24]. In the last years, non-pathogenic live microorganisms, also known as probiotics, have been widely used as immunomodulators to improve protection against pathogens and to preserve mucosal barrier integrity [25]. In recent years, the use of heat-inactivated microorganisms (postbiotics) has been preferred for safety reasons with regards to the most fragile populations, such as the children and elderly [12,26]. The use of postbiotics can be more safe than prebiotics or probiotics in young or immunocompromised individuals for many reasons: (a) in subjects with altered gut mucosa integrity, probiotics might enter into the bloodstream or vital organs causing systemic or localized infections and this risk could increase when the ability of the immune system to patrol is defective; (b) increased release of molecules by probiotics can induce uncontrolled levels of inflammatory mediators and uncontrolled activation of the immune system, thus, promoting autoimmune diseases [12]; (c) prebiotics, used as food by microorganisms, are composed of human milk oligosaccharides, lactulose and inulin derivatives that could induce allergic reactions in hypersensitive subjects [26].

Limited information is available on the effects of postbiotics on macrophage polarization.

The present study was aimed at assessing, for the first time, the immunomodulatory effects of a blend of tyndallyzed (i.e., heat-inactivated) bacteria (TB) composed of three strains of *Lactobacilli* (*Acidofilus*, *Casei* and *Plantarum*) and a strain of *Streptococcus Thermophilus* on macrophage polarization using THP-1-derived macrophages and hMDMs. Data herein presented demonstrate that the tested blend of postbiotics preferentially orientates macrophage polarization toward an M1 phenotype with increasing expression and release of IL-8, IL-1β and IL-6 and reduced levels of TGF-β1. IL-12 gene expression is also induced by the tested blend of TB.

M1 (also known as classically activated) and M2 (also known as alternatively activated) macrophages are differentiated by surface receptors, gene signatures and secretion of inflammatory mediators [2]. Macrophage polarization is a dynamic process by which macrophages can assume pro- or anti-inflammatory functions and contribute to orientate the TH differentiation toward TH1, TH2 or Treg functional T lymphocyte subpopulations [2]. M1 macrophages, which are the prototypic pro-inflammatory macrophage subset, are induced by the exposure to interferon and/or lipopolysaccharide (LPS), while M2, with prominent anti-inflammatory and reparative functions, are induced by TH2 cytokines (IL-4 and IL-13) [21]. Antigen processing and innate immune receptor activation, which lead to higher IFN-gamma and IL-12 levels, favor TH1 polarization, while increased levels of IL-4 favor TH2 polarization and increased IL-10 levels induce Treg activities [27].

Phagocytosis represents an initial event boosting innate immune responses against pathogens. Scavenger receptor activation by Gram-positive or Gram-negative components [28] or by damage-associated molecular patterns (DAMPs) [18] triggers phagocytosis in macrophages. Microbes or apoptotic cells phagocyted upon the activation of scavenger receptors are then exposed to oxygen-dependent and non-dependent attacks finalizing their digestion and destruction [17]. Herein, TB are phagocyted by THP-1-derived macrophages, thus, suggesting the pro-inflammatory activation of these cells.

IL-8 is produced by immunocompetent cells, including macrophages, upon PRRs stimulation and promotes the development of phlogistic events by attracting neutrophils and lymphocytes into the inflammatory sites [29]. Data herein presented show that postbiotics further support M1 polarization by inducing the gene expression and release of IL-8. It has been demonstrated that IL-8 as recombinant or stimulated by microbial products (including lipopolysaccharide, LPS) directly favor pro-inflammatory M1 polarization increasing IL-1β and IL-6 as well as IFN-gamma receptor while reducing IL-4 receptor expression [30].

The degradation of microbes can release other components, for example, DNA of bacteria that can in turn activate cytosolic PRRs that boost the inflammatory responses via inflammasome machinery [31]. IL-1β expression is induced by inflammasome machinery that starts its activation process by the engagement of PRRs. PRRs present on innate immune cells are activated by the binding with PAMPs, derived from microorganisms or by DAMPs derived from tissue damage. IL-1β acts as a relevant bridge between innate and adaptive responses. Monocytes, when stimulated by IL-1β, become dendritic cells, the most potent antigen presenting cells [32]. The blend of postbiotics tested in the present study can increase both the gene expression and the release of IL-1β. These data suggest that the tested blend is also able to induce the upregulation and the processing of pro-IL-1β to mature IL-1β for its release by caspase-1 activation. Following canonical inflammasome activation, mature IL-1β is released in the extracellular space through gasdermin D (GSDMD) pores. Formation of GSDMD pores in the cell membrane is also associated with a lytic proinflammatory form of cell death called pyroptosis. In human monocytes, the so-called “alternative” activation of inflammasome has been reported where IL-1β is processed and released from living cells in the absence of pyroptosis [33]. The finding that TB at the tested concentrations induce IL-1β release in THP1 monocyte-derived macrophages without inducing cell toxicity suggests that they promote the release of IL-1β by the alternative non-pyroptotic mechanism. The different molecular processes associated with the gene expression and synthesis of pro-IL-1 and the release of a mature form could explain why a lower concentration (TB 10^6^) of TB induces the maximal increase in IL-1 beta. It is conceivable that a lower concentration of TB is able to induce higher IL-1β release because higher concentrations of TB inside the macrophage could create a malfunction of the machinery necessary to process and secrete active IL-1β.

IL-6 is a small size glycoprotein produced by innate immune cells, including macrophages, in response to a variety of different stimuli, including allergens, respiratory virus and exercise [34]. IL-6 is a pleiotropic cytokine classically produced by M1 macrophages [20]. IL-6 can reinforce the activity of signals promoting M2 polarization [21]. IL-6 is considered as a pro-inflammatory cytokine and its levels are increased in asthmatics [35]. Moreover, IL-6 in the presence of TGF-β has been reported to induce Th17 cells [34] rather than Treg cells, thus, amplifying inflammatory and immune responses that can be necessary to counteract infections at the early stages [36]. In this regard, it has been demonstrated that anti-cytokine therapy during the initial stages of infection may hinder the development of robust anti-viral T cell responses [37,38]. Herein, we demonstrated that TB are able to induce IL-6 gene expression and release.

PRR activated by PAMPs induces TNF-alpha and PGE2 release [39] as well as IL-12 and IL-10 gene expression [22]. LPS-stimulated monocytes release TNF-alpha within 2 h, with peak values at 8 h and with a decline at subsequent time-points due to PGE2 release [39,40]. PRR activation in the presence of IFN-gamma stimulation leads to IL-12 release, while in the presence of IL-4 and PGE2 it leads to IL-10 release [22]. TB induced the gene expression of IL-12 without detectable protein release in THP-1-derived macrophages. The lack of IFN-gamma stimulation can explain the finding that IL-12 protein release was undetectable in our experimental model. In this regard, the IFN-gamma, derived principally from NK cells and Th1 lymphocytes activated by IL-12 of macrophages or dendritic cells upon pathogen infection, represents a positive feedback mechanism for more robust IL-12 production [22,41].

TGF-β1 is able to reduce scavenger receptors in THP-1 human macrophages [42], thus, limiting phagocytosis and cytokine production in macrophages. In the present study, TB, besides increasing phagocytosis and IL-8, IL-1β and IL-6 expression/release, reduced TGF-β gene expression and protein release, further confirming a prominent effect towards M1 polarization.

TB could also potentially affect adaptive immune response activation. It is known that IL-1β and IL-6 control proliferation, activation and apoptosis of lymphocytes [43], while TGF-β induce Treg activation [27].

Although PMA-stimulated THP-1 cells constitute a suitable model for studying macrophage polarization in vitro [10], previous papers report some limitations regarding the use of THP-1-derived macrophages compared to primary macrophages, including hMDMs.

THP-1-derived macrophages do not express mannose receptor (CD206) or MHC class II DRa1 but express more CD14 and IL-1β compared to primary cells [44]. Compared with primary cells, the response of THP-1 to LPS stimulation [45] is reduced and TLR3 and TLR5 gene expression is lower [46].

To overcome these limitations, we assessed the effect of TB on IL-8, IL-6 and TGF-β1 release in hMDMs. TB induced IL-8 and IL-6 release while they decreased TGF-β1 release in hMDMs, thus, confirming that this blend is able to preferentially induce M1 polarization. IL-1β was undetectable in hMDMs in all the tested experimental conditions. In this regard, it is known that without the addition of an activating stimulus such as ATP or nigericin, the concentration of this cytokine in LPS-stimulated hMDMs remains unchanged [47].

Further future studies are needed to overcome some limitations of the present study and in detail to: (a) confirm the effects of this TB blend on macrophage polarization by also assessing the M1 or M2 phenotype by flow cytometry; (b) demonstrate the ability of this TB blend in reverting M2 toward M1 polarization; (c) assess macrophage polarization effects of TB in vivo directly on allergic patients in dedicated clinical studies.

In conclusion, the study describes new mechanisms by which TB promote M1 polarization in human macrophages. Data herein reported support a role of TB as add-on therapy for the management of diseases, including allergic or infective diseases, which can be ameliorated by an improvement to the M1 polarization process.

## 4. Materials and Methods

### 4.1. THP-1-Derived Macrophage Cultures

The human monocytic cell line THP-1 (ATCC TIB-202, Manassas, VA, USA) was used in this study. The cells were grown in RPMI 1640 medium supplemented with 10% heat-deactivated (56 °C, 30 min) fetal bovine serum (FBS), 1% sodium pyruvate and 2 mM L-glutamine (all from Euroclone, Milan, Italy), without antibiotics. The cell cultures were maintained in a humidified atmosphere of 5% CO_2_ at 37 °C and differentiated in macrophages by adding to the medium 5 ng/mL of phorbol 12-myristate 13-acetate (PMA) (Sigma-Aldrich, St. Louis, MO, USA) for 48 h.

### 4.2. Human Monocyte-Derived Macrophages (hMDMs)

Peripheral blood mononuclear cells (PBMCs) were isolated as previously described [48] from buffy coats derived from healthy subjects and received by ARNAS “Civico, Di Cristina, Benfratelli” (Palermo, Italy) according to a Material Transfer Agreement signed on 6 November 2019.

Human macrophages were obtained by culturing PBMCs for 7 days in a complete RPMI 1640 medium supplemented with 10% FBS and 50 ng/mL of human M-CSF. The medium was replaced after 3 days of culture. The day before each experiment, hMDMs were treated with trypsin-EDTA for 5 min, scraped, plated in complete medium without M-CSF into 6-well plates (1.5 × 10^6^ cells/well) and incubated at 37 °C with 5% CO_2_.

### 4.3. Cell Treatment

On the day of stimulation, the culture medium was changed with a medium containing 1% FBS and without antibiotics. Cells were exposed to or not different concentrations (10^6^, 5 × 10^6^, 10^7^, 5 × 10^7^, 10^8^, 5 × 10^8^, 10^9^ CFU/mL) of a TB blend provided by Stewart Italia and composed of *Lactobacillus Casei*, *Lactobacillus Acidophilus*, *Lactobacillus Plantarum* and *Streptococcus Thermophilus* for different indicated times. Time of incubations were identified on the basis of previous evidence [48,49,50].

### 4.4. Cell Viability Assay

The effect of TB on the cell viability of THP-1-derived macrophages was evaluated by means of the CellTiter 96^®^ Aqueous One Solution Cell Proliferation Assay (Promega, Madison, WI, USA), a colorimetric method capable of determining the number of viable cells by using 3-(4,5-dimethylthiazol-2-yl)-5-(3-carboxy methyl-phenyl)-2-(4-sulfophenyl) 2H-tetrazolium (MTS).

THP-1 cells were seeded in 96-well plates and differentiated in macrophages by PMA treatment for 48 h. After that, THP-1-derived macrophages were treated with different concentrations of TB (10^6^, 5 × 10^6^, 10^7^, 5 × 10^7^, 10^8^, 5 × 10^8^, 10^9^ CFU/mL) for 4 h. At the end of the treatment, 20 µL of CellTiter 96^®^ AQueous One Solution reagent was added to each well and the plates were incubated for 20 min at 37 °C and 5% CO_2_. The absorbance was measured by using a microplate reader at 490 nm (Microplate reader SPECTROstar-Nano, BMG-Labtech, Allmendgrün, Ortenberg, Germany). Results were expressed as the percentage of viability compared with untreated cells (NT) (100% viability).

### 4.5. Staining of TB with SytoRed and Phagocytosis of TB

The bacterial suspension (at a concentration of 10^9^ CFU/mL) was incubated with SytoRed (20 uM, dilution 1:250) (Molecular Probes, Life Technologies, Thermo Fisher Scientific, Waltham, MA, USA) for 30 min at room temperature. After three washes with PBS, the stained bacteria were resuspended in 1 mL of RPMI without FBS and antibiotics. THP-1 cells were seeded in 96-well plates and differentiated for 48 h with PMA. Then, THP-1-derived macrophages were incubated with different concentrations of TB (10^6^, 5 × 10^6^, 10^7^, 5 × 10^7^, 10^8^ CFU/mL) for 30 min. At the end of the incubation, the cells were washed with PBS, fixed in 4% paraformaldehyde for 5 min and washed again with PBS. After that, the cells were stained with Actin Green (Molecular Probes, Life Technologies, Thermo Fisher Scientific, Waltham, MA, USA), which binds actin with high affinity. Nuclei were stained with Hoechst (Molecular Probes, Life Technologies, Thermo Fisher Scientific, Waltham, MA, USA), diluted 1:300 in PBS, as previously described [51], and analyzed by the Operetta CLS system (PerkinElmer, Waltham, MA, USA) in the confocal mode at 63× magnification.

### 4.6. Real-Time PCR

THP-1 cells were seeded in 6-well plates and differentiated in macrophages by PMA treatment for 48 h. THP-1-derived macrophages were treated with different concentrations of TB (10^6^, 5 × 10^6^, 10^7^, 5 × 10^7^, 10^8^ CFU/mL) for 4 h. At the end of the treatment, the whole RNA was isolated using TRIzol Reagent (Life Technologies, Thermo Fisher Scientific, Waltham, MA, USA) following the manufacturer’s instruction. In addition, 1 μg of RNA was reverse-transcribed to cDNA, using the iScript cDNA Synthesis kit (Biorad, CA, USA). IL-8, IL-1β, IL-6, IL-12 and TGF-β1 gene expression was evaluated by qRT-PCR conducted by the Step One Plus Real-time PCR System (Applied Biosystems, Thermo Fisher Scientific, Foster City, CA, USA) using a specific FAM-labeled probe and primers (prevalidated TaqMan Gene expression assay for IL-8, Hs00174103_m1; for IL-1β, Hs01555410_m1; for IL-6, Hs00985639_m1; for IL-12, Hs01011518_m1; for TGF-β1, Hs00998133_m1, Applied Biosystems) as previously described [52,53]. Gene expression was normalized to GAPDH (prevalidated TaqMan Gene expression assay for GAPDH, Hs03929097_g1) as the endogenous control gene. The relative quantification of mRNA was obtained with the comparative Ct method (2^−ΔΔCt^) and was plotted as respective fold-change. Untreated cells (NT) were used as the reference sample.

### 4.7. ELISA

THP-1 cells were seeded in 6-well plates and differentiated in macrophages by PMA treatment for 48 h. THP-1-derived macrophages were treated with different concentrations of TB (10^6^, 5 × 10^6^, 10^7^, 5 × 10^7^, 10^8^ CFU/mL) for 4 h. Additionally, hMDMs were seeded in 6-well plates and treated with different concentrations of TB (10^6^, 5 × 10^7^, 10^8^ CFU/mL) for 4 h. At the end of the stimulation, the release of IL-8, IL-1β, IL-6, IL-12 and TGF-β1 protein in cell supernatants was determined using a human ELISA kit (R&D Systems, Minneapolis, MN, USA) following the manufacturer’s instructions.

### 4.8. Statistical Analysis

Data were expressed as mean ± SD and analyzed by analysis of variance (ANOVA) followed by Bonferroni test. A *p* value < 0.05 was considered to be statistically significant.

## Data Availability

The data used to support the findings of this study are included within the article.

## Abbreviations

IL	interleukin
TGFb	transforming growth factor
M1	classically activated macrophage
M2	alternatively activated macrophages
TB	tyndallized bacteria
hMDMs	human monocyte-derived macrophages
